# The peripheral immune status of granulocytic myeloid-derived suppressor cells correlates the survival in advanced gastric cancer patients receiving cisplatin-based chemotherapy

**DOI:** 10.18632/oncotarget.18297

**Published:** 2017-05-30

**Authors:** Hirokazu Shoji, Kohei Tada, Shigehisa Kitano, Takashi Nishimura, Yasuhiro Shimada, Kengo Nagashima, Kazunori Aoki, Nobuyoshi Hiraoka, Yoshitaka Honma, Satoru Iwasa, Atsuo Takashima, Ken Kato, Narikazu Boku, Kazufumi Honda, Tesshi Yamada, Yuji Heike, Tetsuya Hamaguchi

**Affiliations:** ^1^ Gastrointestinal Medical Oncology Division, National Cancer Center Hospital, Tokyo, Japan; ^2^ Course of Advanced Clinical Research of Cancer, Juntendo University Graduate School of Medicine, Tokyo, Japan; ^3^ Immunotherapy and Cell Therapy Service, St. Lucas International Hospital, Tokyo, Japan; ^4^ Exploratory Oncology Research and Clinical Trial Center, Division of Cancer Immunotherapy, National Cancer Center, Tokyo, Japan; ^5^ Department of Global Clinical Research, Graduate School of Medicine, Chiba University, Chiba, Japan; ^6^ Division of Molecular and Cellular Medicine, National Cancer Center Research Institute, Tokyo, Japan; ^7^ Division of Molecular Pathology, National Cancer Center Research Institute, Tokyo, Japan; ^8^ Division of Chemotherapy and Clinical Research, National Cancer Center Research Institute, Tokyo, Japan

**Keywords:** gastric cancer, peripheral immune status, granulocytic myeloid-derived suppressor cells, chemotherapy, prognosis

## Abstract

**Background:**

The prognostic significance of peripheral immune status in patients with advanced gastric cancer (AGC) remains unclear.

**Results:**

From July 2013 through December 2014, 37 patients were enrolled. Among patients with 25 subsets of immune cells, patients in the high group of granulocytic myeloid-derived suppressor cells (Gr-MDSCs) showed significantly shorter progression-free survival (PFS) than those in the low group (3.98 vs. 8.78 months; hazards ratio (HR), 2.61; *p* = 0.01). In multivariate analysis, the high Gr-MDSCs value was also associated with shorter PFS (HR, 4.60; 95% confidence interval (CI), 1.79−11.8; *p* = 0.001). Although significant difference was not found in univariate analysis, the high Gr-MDSCs group was associated with shorter overall survival (OS) (HR, 2.89; 95% CI, 1.23–6.80; *p* = 0.015) in multivariate analysis.

**Materials and Methods:**

In this explorative prospective study, peripheral blood samples were collected from AGC patients before initiating first-line cisplatin-based chemotherapy (S-1 + cisplatin or S-1 + cisplatin + docetaxel). Peripheral blood mononuclear cells were analyzed for 25 immune subsets by multicolor flow cytometry. PFS and OS were compared between the patients divided into high and low (≥ and < median, respectively) groups based on the median value for each immune cell subset.

**Conclusions:**

The peripheral immune status of Gr-MDSCs appears to affect the prognosis in AGC. Further research is needed to confirm the clinical value of the level of circulating Gr-MDSCs as a prognostic and/or predictive marker in AGC.

## INTRODUCTION

Almost one million new cases of gastric cancer were estimated to have occurred in the world in 2012, making it the fifth most common cause of cancer-related death, though its incidence is declining in developed countries [1]. In Japan, gastric cancer is the first most common malignant disease in men and the third in women. While the mortality rate of gastric cancer has been continuously decreasing, it still shows the second highest mortality rate [2]. Systemic chemotherapy is of crucial importance for advanced gastric cancer (AGC) patients, in order to obtain palliation of symptoms and improve survival. However, the prognosis for patients with AGC remains unsatisfied with median survival times of 10–13 months [3, 4].

Immune cells respond to tumor cells and eliminate them in cancer patients. On the other hand, some study showed that the immune system could also prompt tumor progression [5–7]. Therefore, the balance between activation and suppression of immune responses may determine whether cancers avoid detection by immune recognition.

Myeloid-derived suppressor cells (MDSCs), one of the representative immune suppressive cells, are a heterogeneous population of myeloid lineage affecting immature state and having the capacity to suppress T-cell responses. Human MDSCs are classified into CD15^+^ granulocytic MDSCs (Gr-MDSCs) or CD14^+^ monocytic MDSCs (M-MDSCs). As for functional differences, Gr-MDSCs are known to express high levels of arginase and reactive oxygen species (ROS), whereas M-MDSCs express both arginase and inducible nitric oxide synthetase but do not produce high levels of ROS [8]. Several suppressive functions of MDSCs have been suggested such as inhibition of T cell (CD4^+^ and CD8^+^) activation, and increase T cell apoptosis [9, 10]. Recent studies have shown that circulating MDSCs are significantly increased in cancer patients and correlate with clinical cancer stages and metastatic disease [11]. In addition, several studies reported that tumor tissues with high MDSCs infiltration are related to poor prognosis and resistance to various therapies [8, 12].

FoxP3^+^ T regulatory cells (Tregs), other immune suppressive cell, accumulate in tumors and increase in the peripheral blood of cancer patients, and the increased Tregs in tumors frequency has been shown to be a marker of poor prognosis in various types of cancers [13, 14].

By contrast, cytotoxic CD4^+^ or CD8^+^ T cells recognize tumor-specific antigen and tumor-associated antigen and exert direct cytotoxic actions against tumor cells. Among the effector T cells, effector memory T cells play an important role for anti-tumor immunity [15, 16].

To date, many retrospective studies have shown the association between these immune cells infiltration and prognosis in various types of tumors. For gastric cancer, several studies have reported a positive association between the extent of the tumor-infiltrating lymphocyte (TIL) infiltrate and a prognosis [17–19]. However, because the limited opportunities for obtaining resected specimens in AGC patients and TIL analyses are difficult to conduct, alternative immunological parameters in more easily assessable samples for predicting prognosis are needed. The association between peripheral immune status and a prognosis in patients with AGC has not yet been fully cleared to the present time. Thus, we conducted prospective study to quantify 25 immune cell subsets of the lymphocytes in the peripheral blood, and investigate their prognostic impacts in patients with AGC receiving cisplatin-based first-line chemotherapy.

## RESULTS

### Patient characteristics

The baseline characteristics of the 37 patients are summarized in Table 1. All patients had good performance status (PS, 0–1). Of 37 patients, 28 (75.7%) were diffuse-type. Twenty-two (59.5%) patients were treated with cisplatin and S-1 (CS), and 15 (40.5%) were treated with docetaxel, cisplatin and S-1 (DCS) (Table 1). A total of 30 (81.0%) patients received second-line chemotherapy (Supplementary Tables 1 and 2).

**Table 1:** Patient characteristics

	*N* = 37	%
Age, median (range)	63	(26–79)
Sex		
Male	18	48.6
Female	19	51.4
Performance status		
0	10	27.0
1	27	73.0
Disease status		
Stage IV	25	67.6
Recurrence	12	32.4
Histology		
Intestinal type	9	24.3
Diffuse type	28	75.7
Target lesion		
Yes	21	56.8
No	16	43.2
Number of metastatic sites		
1	20	54.1
2 ≥	17	45.9
HER2 status		
Positive	3	8.1
Negative	34	91.9
ALP		
≤ 359	28	75.7
> 360	9	24.3
Treatment regimen		
S−1+Cisplatin	22	59.5
Docetaxel+Cisplatin+S−1	15	40.5

ALP, alkaline phosphatase.

**Table 2:** Association between the quantity of each immune subset and progression-free survival

Factors		Hazard ratio^*^	95% CI	*p*-value
Suppressor cells	Granulocytic MDSCs	2.61	1.20–5.68	0.01
	Monocytic MDSCs	0.88	0.43–1.82	0.73
	CD4^+^FOXP3^+^	0.64	0.32–1.26	0.19
	Naïve Tregs	1.00	0.51–1.97	0.99
	Effector Tregs	1.01	0.51–2.00	0.98
Effector cells	CD3^+^	0.79	0.39–1.57	0.49
	CD4^+^	1.03	0.52–2.04	0.93
	Naïve	0.78	0.39–1.56	0.48
	Central memory	1.56	0.79–3.07	0.20
	Effector memory	0.87	0.44–1.72	0.69
	Terminally differentiated effector	0.62	0.31–1.23	0.17
	Granzyme B^+^	1.14	0.57–2.27	0.71
	Perforin^+^	0.99	0.50–1.96	0.99
	Ki67^+^	1.11	0.56–2.18	0.77
	CD8^+^	0.80	0.40–1.59	0.52
	Naïve	1.20	0.61–2.36	0.61
	Central memory	0.74	0.38–1.46	0.39
	Effector memory	0.65	0.33–1.28	0.21
	Terminally differentiated effector	0.65	0.33–1.29	0.21
	Granzyme B^+^	0.75	0.38–1.49	0.41
	Perforin^+^	0.85	0.43–1.68	0.65
	Ki67^+^	0.98	0.50–1.95	0.96
	Natural killer	0.78	0.39–1.53	0.47
Antigen presenting cells	Myeloid dendritic cells	0.98	0.47–2.04	0.96
	Plasmacytoid dendritic cells	0.83	0.40–1.71	0.61

MDSCs, myeloid-derived suppressor cells; Tregs, regulatory T cells.

^*^ Low group (< median) in each immune subset was used as a reference.

### Clinical course

Median follow-up period was 19.6 months (range; 1.7–22.5). 27 (73.0%) of the 37 patients had died. The median survival time (MST) was 11.7 months (95% CI 8.85–14.2). The median progression-free survival (PFS) was 5.6 months (95% CI 5.56–7.47). No adverse events leading to treatment discontinuation were observed in any patients.

### Associations between the quantity of each immune cell subset and PFS

**Figure 1:**
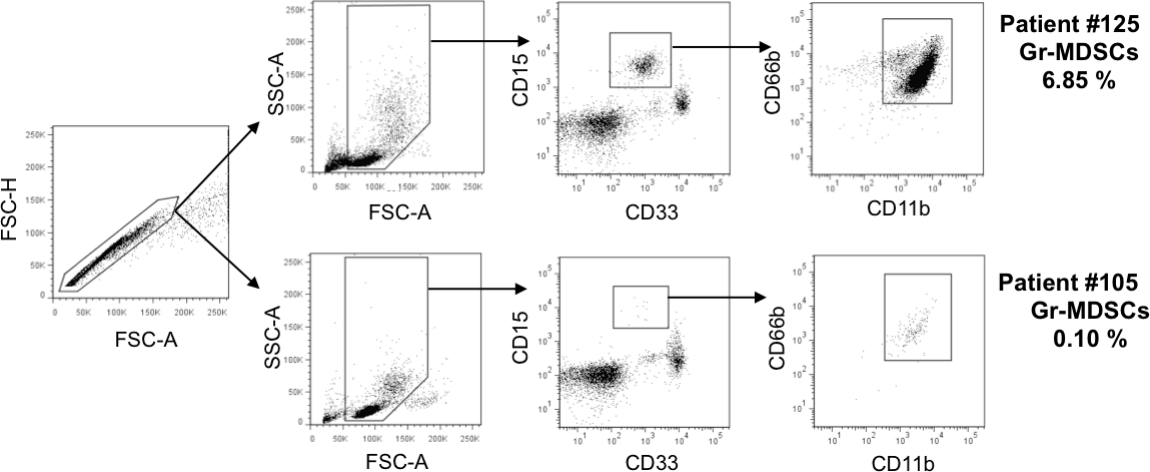
Gating strategy and representative dot plots for granulocytic myeloid-derived suppressor cells (Gr-MDSCs) Two independent dot plots are shown as high and low Gr-MDSCs.

An illustration of the gating strategy and representative dot plots for Gr-MDSCs is shown in Figure 1. The median value of the proportion for Gr-MDSCs was 0.420 % (range, 0.089–7.366). The gating strategy and representative dot plots for other immune cell subsets (M-MDSCs, Tregs, CD4^+^ or CD8^+^ T, Dendritic cell (DC), NK cells, Granzyme B+, Perforin+, or Ki-67+ cells) are shown in Supplementary Figure 1.

**Figure 2:**
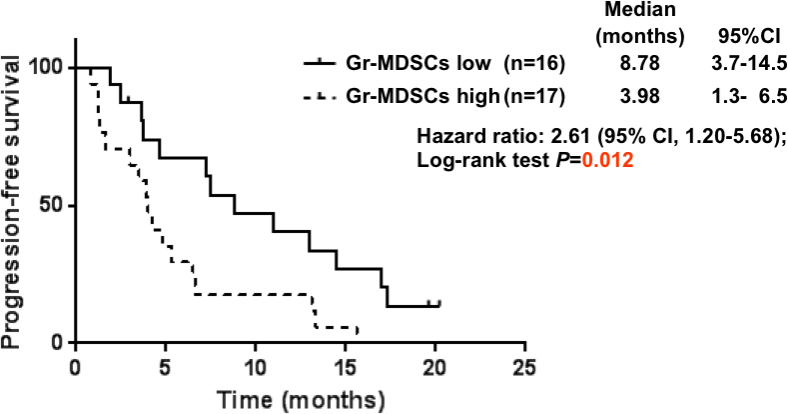
Progression-free survival curves calculating using the Kaplan-Meier methods for groups classified according to the pretreatment proportion of granulocytic myeloid-derived suppressor cells

Of 37 patients enrolled into this study, analysis of MDSCs was performed in 33 patients. Patients were divided into high (≥ median) and low (< median) groups based on the median value of the proportion for each immune cell subset, and PFS was then compared between each pair of groups (Table 2). The median PFS among patients with Gr-MDSCs high group (4.0 months) was significantly shorter than the median PFS among those with Gr-MDSCs low group (8.8 months) (HR, 2.61; 95%CI, 1.20–5.68; p=0.012; Table 2 and Figure 2). Multivariate analyses demonstrated that the proportion of Gr-MDSCs (HR, 4.60; 95% CI, 1.79—11.8 for high group vs. low group), having target lesion (HR, 3.43; 95% CI, 1.43—8.22 for yes vs. no), and treatment regimen (HR, 2.77; 95% CI, 1.13—6.80 for DCS regimen vs. CS regimen) were associated with a shorter PFS (Table 3).

**Table 3:** Univariate and multivariate Cox regression analyses for progression-free survival

Covariates	Univariate			Multivariate		
	HR	95% CI	*P*–value	HR	95% CI	*P*–value
Gr-MDSCs						
Low	Reference			Reference		
High	2.61	1.20–5.68	0.012	4.60	1.79–11.8	0.001
Age						
< 65	Reference					
≥ 65	0.66	0.30–1.44	0.294			
Sex						
Male	Reference					
Female	0.93	0.45–1.93	0.846			
Performance status						
0	Reference					
1	0.69	0.32–1.48	0.341			
Disease status						
Recurrence	Reference					
Stage IV	1.16	0.55–2.45	0.704			
Histology						
Intestinal type	Reference					
Diffuse type	1.30	0.55–3.10	0.548			
Target lesion						
No	Reference			Reference		
Yes	2.16	1.02–4.60	0.045	3.43	1.43–8.22	0.006
Number of metastatic sites						
1	Reference					
≥ 2	0.79	0.38–1.63	0.522			
HER2 status						
Negative	Reference					
Positive	1.49	0.20–11.1	0.696			
ALP						
≤ 359	Reference					
> 360	1.26	0.57–2.77	0.573			
Treatment regimen						
Cisplatin+S−1	Reference			Reference		
Docetaxel+Cisplatin+S−1	1.44	0.68–3.04	0.341	2.77	1.13–6.80	0.026

Gr-MDSCs, granulocytic myeloid-derived suppressor cells; ALP, alkaline phosphatase.

### Associations between the quantity of each immune cell subset and overall survival (OS)

**Figure 3:**
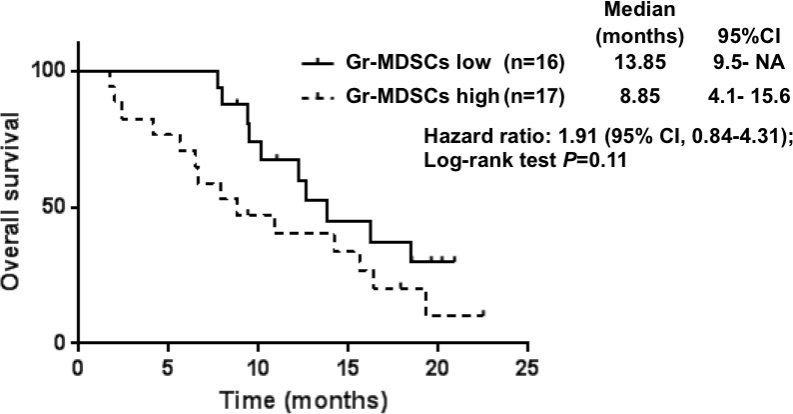
Overall survival curves calculating using the Kaplan-Meier methods for groups classified according to the pretreatment proportion of granulocytic myeloid-derived suppressor cells

Associations between the quantity of each immune cell subset (divided to high and low groups by its median value) and OS are shown in the Table 4. The MST was 8.9 months and 13.9 months in the patients with Gr- MDSCs high and in those with Gr-MDSCs low (*p* = 0.114; Figure 3), respectively.

**Table 4:** Association between the quantity of each immune subset and overall survival

Factors		Hazard ratio^*^	95% CI	*p*-value
Suppressor cells	Granulocytic MDSCs	1.91	0.84–4.31	0.12
	Monocytic MDSCs	1.01	0.45–2.26	0.99
	CD4^+^FOXP3^+^	0.89	0.42–1.89	0.76
	Naïve Tregs	0.79	0.37–1.66	0.53
	Effector Tregs	0.94	0.44–1.97	0.86
Effector cells	CD3^+^	0.61	0.28–1.30	0.20
	CD4^+^	1.58	0.75–3.35	0.23
	Naïve	0.72	0.33–1.54	0.39
	Central memory	1.56	0.73–3.31	0.25
	Effector memory	0.96	0.46–2.04	0.92
	Terminally differentiated effector	0.66	0.31–1.40	0.28
	Granzyme B^+^	1.88	0.86–4.09	0.11
	Perforin^+^	1.09	0.51–2.32	0.82
	Ki67^+^	1.11	0.52–2.33	0.79
	CD8^+^	0.67	0.32–1.43	0.30
	Naïve	0.60	0.28–1.30	0.20
	Central memory	0.92	0.44–1.96	0.83
	Effector memory	0.72	0.34–1.53	0.40
	Terminally differentiated effector	0.91	0.43–1.92	0.81
	Granzyme B^+^	1.01	0.48–2.12	0.98
	Perforin^+^	1.06	0.50–2.22	0.89
	Ki67^+^	0.76	0.36–1.62	0.48
	Natural killer	0.98	0.46–2.10	0.96
Antigen presenting cells	Myeloid dendritic cells	0.69	0.31–1.56	0.37
	Plasmacytoid dendritic cells	0.64	0.28–1.43	0.28

MDSCs, myeloid-derived suppressor cell; Treg, regulatory T cells.

^*^ Low group (< median) in each immune subset was used as a reference.

The results of the univariate and multivariate analyses are summarized in Table 5. Multivariate analyses demonstrated that the proportion of Gr-MDSCs (HR, 2.89; 95% CI, 1.23—6.80 for high group vs. low group), disease status (HR, 2.81; 95% CI, 1.07—7.36 for stage IV vs. recurrence), and having target lesion (HR, 2.89; 95% CI, 1.12—7.44 for yes vs. no) were associated with a poor prognosis.

**Table 5:** Univariate and multivariate Cox regression analyses for overall survival

	Univariate			Multivariate		
	HR	95% CI	*P*–value	HR	95% CI	*P*–value
Gr-MDSCs						
Low	Reference			Reference		
High	1.91	0.84–4.31	0.114	2.89	1.23–6.80	0.015
Age						
< 65	Reference					
≥ 65	0.79	0.34–1.86	0.592			
Sex						
Male	Reference					
Female	1.03	0.45–2.29	0.949			
Performance status						
0	Reference					
1	1.67	0.66–4.23	0.276			
Disease status						
Recurrence	Reference			Reference		
Stage IV	2.44	0.96–6.22	0.062	2.81	1.07–7.36	0.035
Histology						
Intestinal type	Reference					
Diffuse type	1.98	0.66–5.92	0.220			
Target lesion						
No	Reference			Reference		
Yes	2.27	0.98–5.28	0.057	2.89	1.12–7.44	0.028
Number of metastatic sites						
1	Reference					
≥ 2	1.42	0.63–3.19	0.396			
HER2 status						
Negative	Reference					
Positive	1.64	0.22–12.41	0.631			
ALP						
≤ 359	Reference					
> 360	1.50	0.65–3.44	0.344			
Treatment regimen						
Cisplatin+S−1	Reference					
Docetaxel+Cisplatin+S−1	0.97	0.41–2.29	0.948			

Gr-MDSCs, granulocytic myeloid-derived suppressor cells; ALP, alkaline phosphatase.

### Changes in proportional of Gr-MDSCs during chemotherapy

We investigated the relationship between the changes in proportion of Gr-MDSCs during 1st-line systemic chemotherapy and prognosis. 24 samples at 2nd blood collection were available. The means of % Gr-MDSCs was compared between before (1st blood collection) and after chemotherapy (2nd blood collection) using a paired-t test; however, there was no tendency for changes of %Gr-MDSCs (Supplementary Figure 2A). When dichotomized based on increase or decrease in the proportion of Gr-MDSCs from baseline versus, there were no correlation with OS (Supplementary Figure 2B).

### Immune status proportion of Gr-MDSCs at discontinuing chemotherapy

We next assessed the change of 7 samples, which could be collected at progression. The changes in proportion of Gr-MDSCs at the time of disease progression from baseline are presented in Table 6. Among the 5 patients with high Gr-MDSCs group at baseline, 4 had a remarkable elevation of the proportion of Gr-MDSCs at disease progression, and had extremely poor survival time. In the patient (no. 33), the proportion of Gr-MDSCs at PD was decreased to 0.610 % from 0.884 %. Interestingly, OS time of the patient was 22.5 months, and he continued to receive subsequent chemotherapy at data cut off.

**Table 6:** Changes in the proportion of Gr-MDSCs (%) from baseline and the time of 1st-line progression

Patient No	Regimen	Baseline group	Proportion of Gr-MDSCs (%)			OS (months)	PFS (months)	Second line therapy
			Baseline	1st-line PD or 6 months after initiating chemotherapy (whichever comes first)				
47	DCS	low	0.258	0.148	PD	11.0 (c)^**^	1.9	CPT-11
35	CS	low	0.405	1.223	PD	13.8	4.6	CPT-11
77	DCS	high	0.742	6.928	PD	2.0	1.3	PTX
33	DCS	high	0.884	0.610	PD	22.5 (c)^*^	6.6	CPT-11
34	CS	high	0.917	1.555	PD	2.4	1.6	PTX
55	CS	high	1.157	2.143	PD	5.6	4.0	PTX
30	CS	high	2.582	8.789	PD	6.5	5.3	−

Gr-MDSCs, granulocytic myeloid-derived suppressor cells; DCS, docetaxel, cisplatin, plus S-1; CS, cisplatin plus S-1; PD, progressive disease.

OS, overall survival; PFS, progression-free survival; CPT-11, irinotecan; PTX, paclitaxel; (c), censored case; ^*^, ongoing treatment at data cut off.

^**^, lost to follow up.

### Comparison of plasma cytokine concentration according to Gr-MDSCs subgroups

In order to determine whether there is a difference in cytokine concentrations according to Gr-MDSCs subgroups, 8 cytokines in plasma were measured and compared (Supplementary Table 3). The IL-8 level in the Gr-MDSCs high group was significantly higher than that in the Gr-MDSCs low group (mean ± SD, 22.27 ± 11.72 vs. 12.61 ± 6.03; *p* = 0.01). There were no significant differences in other cytokines, such as IFN-γ, IL-1β, IL-4, IL-6, IL-10, IL-12p70, and TNF-α, between these two groups.

We also analyzed the association between cytokine concentrations and survival (Supplementary Tables 4 and 5). Moreover, we performed multivariate analyses that included cytokines (IL-6 and IL-8 were statistically significant in univariate analyses) in the final models (Tables 3 and 5) as sensitivity analyses (data not shown). In this sensitivity analyses, the peripheral immune status of Gr-MDSCs consistently appears to affect the prognosis in AGC.

## DISCUSSION

In this prospective observational study, we analyzed 25 immune cell subsets status, and investigated whether immune cell subsets affected the survival in advanced gastric cancer patients. We found that the high proportion of Gr-MDSCs before chemotherapy was poor prognostic factor for PFS in AGC patients who received cisplatin-based chemotherapy, and tended to be correlated with shorter OS, suggesting that this characteristic might serve as a prognostic factor. This study suggested that immune status may affect the efficacy of chemotherapy in AGC patients.

Many studies reported a survival benefit associated with the presence of TIL [17–22], including gastric cancer. There are very few studies addressed in the clinical significance of MDSCs in gastric cancer tissues. Choi et al. reported the prognostic effects of tumor infiltrating MDSCs. They investigated the frequencies of TILs in 28 surgically resected gastric cancer tissues by using flow cytometry, and showed an increased proportion of MDSCs in tissues was a poor prognostic factor [23]. However, in their study, the association between MDSCs status in peripheral blood and tumor tissues was not investigated. Although information obtained from surgical specimens appear extremely useful, however, TIL analyses are difficult to conduct, giving the limited opportunities for obtaining sufficient specimens in AGC patients. Therefore, we studied the immune cell subsets in peripheral blood samples which were easily obtained with minimally invasive procedure.

In cancer patients, MDSCs in the peripheral blood increase substantially [24–26]. Correlations between circulating number of MDSCs in various types of cancers including gastric cancer and survival have been investigated in two studies so far. Gabitass et al. reported that the high percentage of MDSCs were an independent prognostic factor in patients with pancreatic, esophageal, and gastric cancer [27]. In their report, 25 non-cardia gastric cancer patients were included. Of these, 17 patients were stage IV disease. Wang et al. also found that gastric cancer patients with high MDSCs exhibited a significantly shorter survival time compared with patients with low MDSCs [28]. In the Wang's study using the similar definition of MDSC subtype to the present study, there was no difference in OS between the high and low groups. The discrepancy between the Wang's and this study was considered to be caused by the difference in the study population; stage I-IV cases in the Wang's study and stage IV or recurrent cases in this study.

We assessed how the change in proportion of Gr-MDSC during 1st-line chemotherapy affected OS. Of 37 patients, we could obtained 7 paired pretreatment and 1st-line PD blood samples. In the low group of Gr-MDSCs (*n* = 2), all patients had comparatively low proportion of Gr-MDSCs at PD, and they had favorable long-term survival. On the other hand, in the high group of Gr-MDSCs at baseline, most patients had high proportion of Gr-MDSCs at PD and they had short-term survival. Interestingly, one of five who showed decrease in Gr-MDSCs compared to pretreatment samples, survived long. Preclinical works have suggested that two chemotherapeutic drugs, gemcitabine and 5-fluorouracil (5-FU), deplete MDSCs. As compared to gemcitabine, 5-FU induced a more potent apoptosis-mediated MDSC depletion *in vitro* and *in vivo* [29, 30]. Indeed S-1, an oral prodrug of 5-FU, administrated in all patients in our study, that might relate to the changes of MDSCs numbers. Although we could analyze only limited patient samples in high group of Gr-MDSCs, it is speculated that the decrease in Gr-MDSCs level before starting 2nd-line chemotherapy might occur among the rest cases without blood samples available, which could be one of the reasons for not having statistical significance difference in OS between two groups.

We also evaluated plasma cytokines that affect the formation of immune cell subsets. Among eight cytokines, IL-8 was significantly higher in the Gr-MDSCs high group than in the Gr-MDSCs low group. Previous papers showed that IL-8 was contributed to gastrointestinal carcinogenesis through increased recruitment of MDSCs [31]. Furthermore, increased levels of circulating MDSCs were associated with increasing advanced disease stage, increased serum IL-8 levels, and defective T-cell function [32]. From these findings, we suggest that increased IL-8 in our cohort may have contributed to the adverse immune status.

This study has some limitations. First, our study consisted of small number of samples. This resulted in statistical underpowered and limited our ability to identify statistically significant differences among the immune cell subsets. Therefore, the results need to be validated in a prospective manner with sufficient sample size. Second, the possibility that the high population of diffuse-type in our study may affect the immune status cannot be completely excluded. Despite these limitations, our study represents the largest flow cytometric characterization of peripheral immune cell subsets in AGC published so far.

In summary, we explored 25 immune cell subsets in peripheral blood from patients with unresectable AGC before first-line cisplatin-based chemotherapy and identified high proportion of Gr-MDSCs as significant adverse factors for PFS. Furthermore, high proportion of Gr-MDSCs at pretreatment tended to result in a shorter survival period, compared to cases with low proportion of Gr-MDSCs. Further research on a large population is needed in order to validate our results in gastric cancer as well as other types of cancer.

## MATERIALS AND METHODS

### Patients

Patients with AGC who received systemic first-line systemic chemotherapy between July 2014 and December 2015 at National Cancer Center Hospital were prospectively enrolled.

The eligibility criteria for this study were as follows: 20 years or older; histological proven primary gastric adenocarcinoma; scheduled to receive cisplatin-based first-line chemotherapy (CS regimen or DCS regimen); no active viral infection such as with the human immunodeficiency virus, hepatitis B and/or C. The CS regimen consisted of infusion of cisplatin (60 mg/m^2^/day) on day 8 and oral S-1 (80 mg/m^2^/day) for 3 weeks on days 1–21 followed by a 2-week rest. The DCS regimen consisted of infusion of docetaxel (40 mg/m^2^/day) and cisplatin (60 mg/m^2^/day) on day 1, and oral S-1 (80 mg/m^2^/day) for 2 weeks on days 1–14 followed by a 2-week rest. These treatments were repeated until disease progression, appearance of unacceptable toxicities or patient's refusal. To evaluate response, systemic computed tomography was repeated every two months.

The following characteristics of patients were collected: age, Eastern Cooperative Oncology Group performance status (ECOG PS), histology, history of gastrectomy, metastatic sites, presence of target lesion according to the response evaluation criteria in solid tumors (RECIST) version 1.1, serum alkaline phosphatase (ALP) level. All of the patients provided informed consent for study registration and blood collection. The study protocol was reviewed and approved by the institutional ethics committee of the National Cancer Center and St. Luke's International Hospital.

### Staining of peripheral blood mononuclear cells

Peripheral blood was collected within 5 days before initiating chemotherapy. The second was performed just before the 3rd cycle (11th week) in the CS cohort or the 3rd cycle (9th week) in the DCS cohort, respectively, and 3rd collection was done at 6 months after initial chemotherapy in both cohorts. In case dicontinuing chemotherapy from any reason before the 2nd or 3rd blood collection, blood was collected at the time of discontinuation. Peripheral blood mononuclear cells (PBMCs) were isolated from peripheral blood by density gradient centrifugation. MDSCs was measured in fresh PBMCs immediately after collection because previous study demonstrated that a part of MDSCs was decreased by cryopreservation [33]. DC was measured at the same time as measurement of MDSCs because common panel was used for both subsets. The rest of PBMCs were cryopreserved and used for measurement of T cells, Tregs, and NK cells.

For staining of intracellular protein (Ki-67, FOXP3, perforin, and granzyme B), FOXP3/Transcription Factor for Staining Buffer Set (eBioscience) was used according to the manufacture's protocol. 2.5–5 × 10^5^ PBMCs were suspended with 100 μL staining buffer (PBS containing 2% fetal bovine serum, FBS). The antibodies for surface markers were then added for 30 minutes at 4°C. Used antibodies were as follows; Lineage (Lin, CD3/CD16/CD19/CD20/CD56) cocktail FITC, CD14-PerCP-Cy5.5, CD11b-APC-Cy7, CD33-PE-Cy7, CD11c-Alexa Fluor700, CD123-Brilliant Violet 421, CD15-V500, CD3-APC, Ki-67-Alexa Fluor700, CD8-APC-Cy7, Granzyme B-FITC, CD56-PE-CF594 (BD Pharmingen), CD4-Brilliant Violet 650, CD16-PerCP-Cy5.5, CCR7-PerCP-Cy5.5 (Biolegend), FOXP3-PE, CD66b-APC (eBioscience), CD45RA-FITC, HLA-DR-ECD (Beckman Coulter), Perforin-PE (cell sciences). Isotype controls included the appropriate fluorochrome-conjugated mouse IgG1, IgG 1k, or IgG2a k (BD Pharmingen, Biolegend, eBioscience). The stained cells were detected using a LSR II Fortessa with FACS Diva software (BD Biosciences). All analyses were carried out using FlowJo software (Tree star).

### Definition and analysis of immune subsets

Twenty-five immune subsets were analyzed in this study. Definitions of immune subsets were as follows; Gr-MDSCs, CD33^dim^CD15^+^CD66^+^CD11b^+^: M-MDSCs, Lin^−^CD14^+^CD33^+^CD11b^+^HLA-DR^low/-^: naive Tregs: CD3^+^CD4^+^CD45RA^+^FOXP3^low^: effector Tregs, CD3^+^CD4^+^CD45RA^-^FOXP3^high^: plasmacytoid DC, Lin^−^CD14^−^CD123^+^HLA-DR^high^: myeloid DC, Lin^−^CD14^−^CD11c^+^HLA-DR^high^. T cells were classified as naïve (CD45RA^+^CCR7^+^), central memory (CD45RA^−^CCR7^+^), effector memory (CD45RA^−^CCR7^−^), and terminally differentiated effector cells (CD45RA^+^CCR7^−^) in CD4^+^ or CD8^+^ cells. The expression levels of granzyme B, perforin, and Ki-67 were also assessed in CD4^+^ or CD8^+^ T cells. Appropriate isotype controls were used for cut-off between positivity and negativity.

The proportion of lymphoid subsets was obtained by dividing cell number of each subset by cell number of lymphocyte fraction based on the result from flow cytometry analysis. The proportion of Gr-MDSCs was calculated by dividing the cell number of CD33^dim^CD15^+^CD66^+^CD11b^+^ cells by the number of PBMCs.

### Measurement of cytokines in plasma

Cryopreserved plasma was used for measuring interferon (IFN)-γ, interleukin (IL)-1β, IL-4, IL-6, IL-8, IL-10, IL-12p70, and tumor necrosis factor (TNF)-α. These cytokines were simultaneously measured on an MSD SECTOR Imager 2400 instrument (Meso Scale Discovery, Inc) according to the manufacturer's protocol.

### Statistical analysis

Differences between categorical variables were assessed using the Fisher's exact tests. Based on the median value of proportion for each immune cell subset, the patients were divided into high (≥ median) and low (< median) groups. The Mann-Whitney *U* test was used for comparisons of measured values of cytokines. PFS was defined as the time from initiation of chemotherapy until detection of progression. Patients who were lost to follow-up were treated as censored observations at the last contact. The OS period was defined as the time from initiation of chemotherapy until the date of death or the latest follow-up. Patients who were lost to follow-up were treated as censored cases at the last confirmation of survival. PFS and OS curves were calculated using the Kaplan-Meier method, and compared with the log-rank test. The median follow-up time for survival was calculated by means of the reverse Kaplan–Meier method. For univariate and multivariate analyses, the Cox proportional regression model was used.

All *P* values are two-sided. *P* values of < 0.05 were considered to indicate statistical significance, and 95 % confidence intervals were calculated. All statistical analyses were performed using SAS software version 9.4 (SAS Institute, Cary, NC, USA), and Graphpad Prism 5 software package.

## SUPPLEMENTARY MATERIALS FIGURES AND TABLES


