# Supplementary material for: The peripheral immune status of granulocytic myeloid-derived suppressor cells correlates the survival in advanced gastric cancer patients receiving cisplatin-based chemotherapy
Source: Oncotarget. 2017 May 30;8(56):95083–94. doi: 10.18632/oncotarget.18297 (PMC5707007; doi:10.18632/oncotarget.18297)
Supplement: Supplementary file 1 [file oncotarget-08-95083-s001.pdf]

The peripheral immune status of granulocytic myeloid-derived suppressor cells correlates the survival in advanced gastric cancer patients receiving cisplatin-based chemotherapy

Supplementary Materials

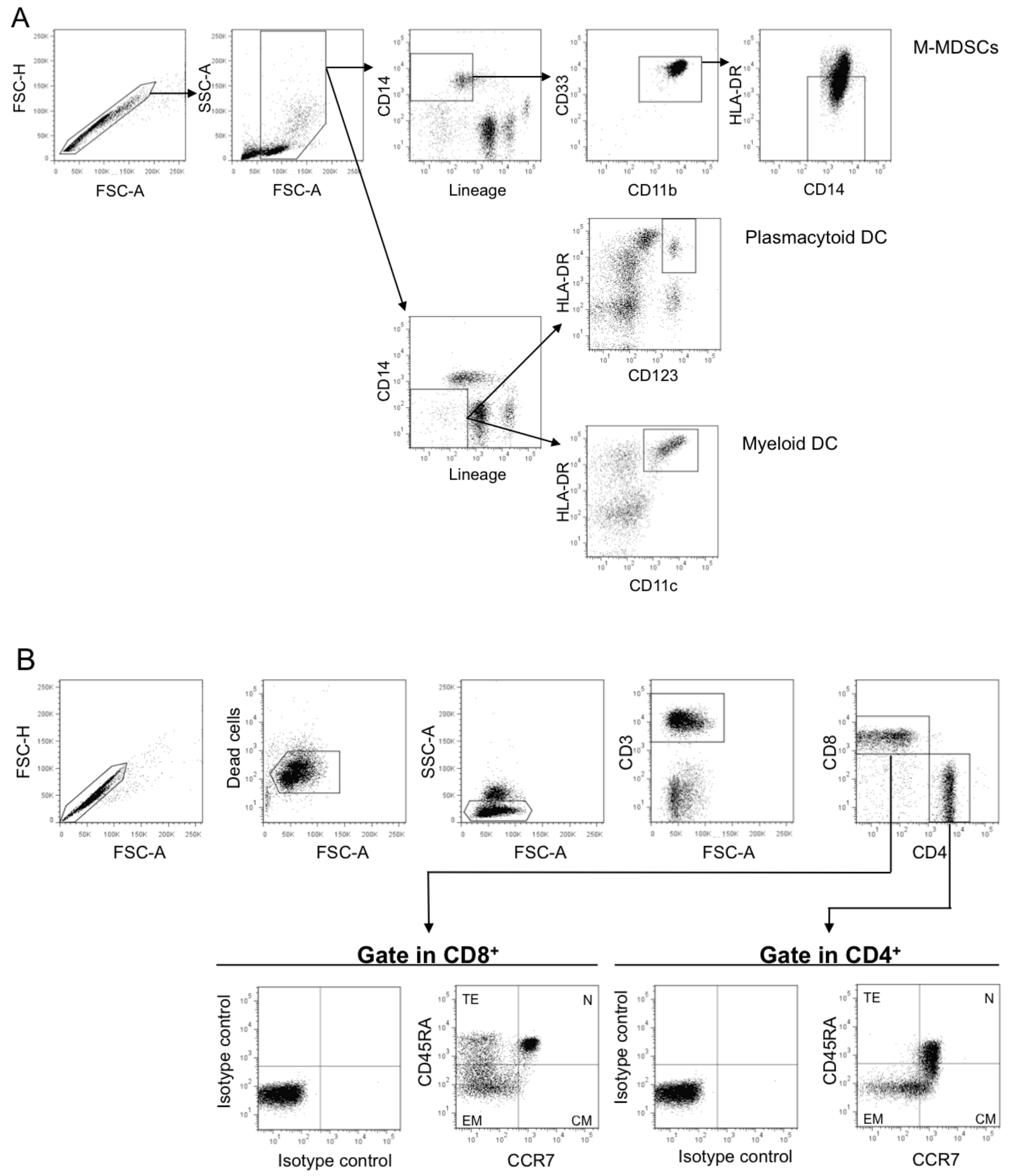

C

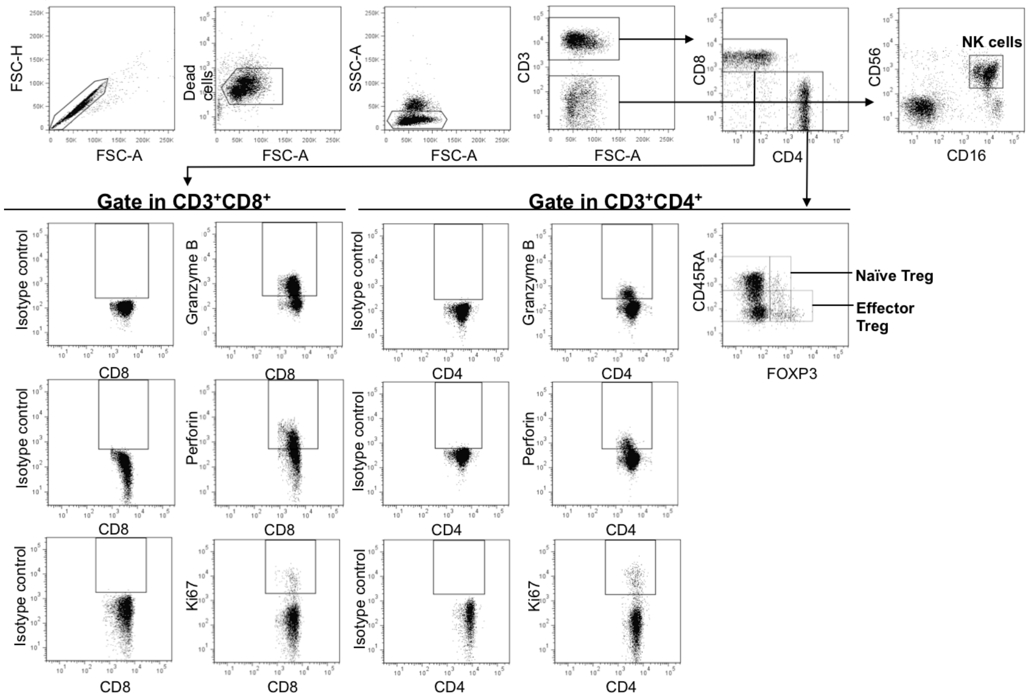

Supplementary Figure 1: Gating strategy and representative dot plots for M-MDSCs, Plasmacytoid DC, Myeloid DC (**A**), naïve/ memory cells in T cell fractions (**B**), and Treg, NK, Granzyme B+, Perforin+ and Ki-67+ cells (**C**). M-MDSCs, monocytic myeloid-derived suppressor cells; DC, dendritic cell; N, naïve; CM, central memory; EM, effector memory; TE, terminally differentiated effector; Tregs, regulatory T cells; NK, natural killer cell.

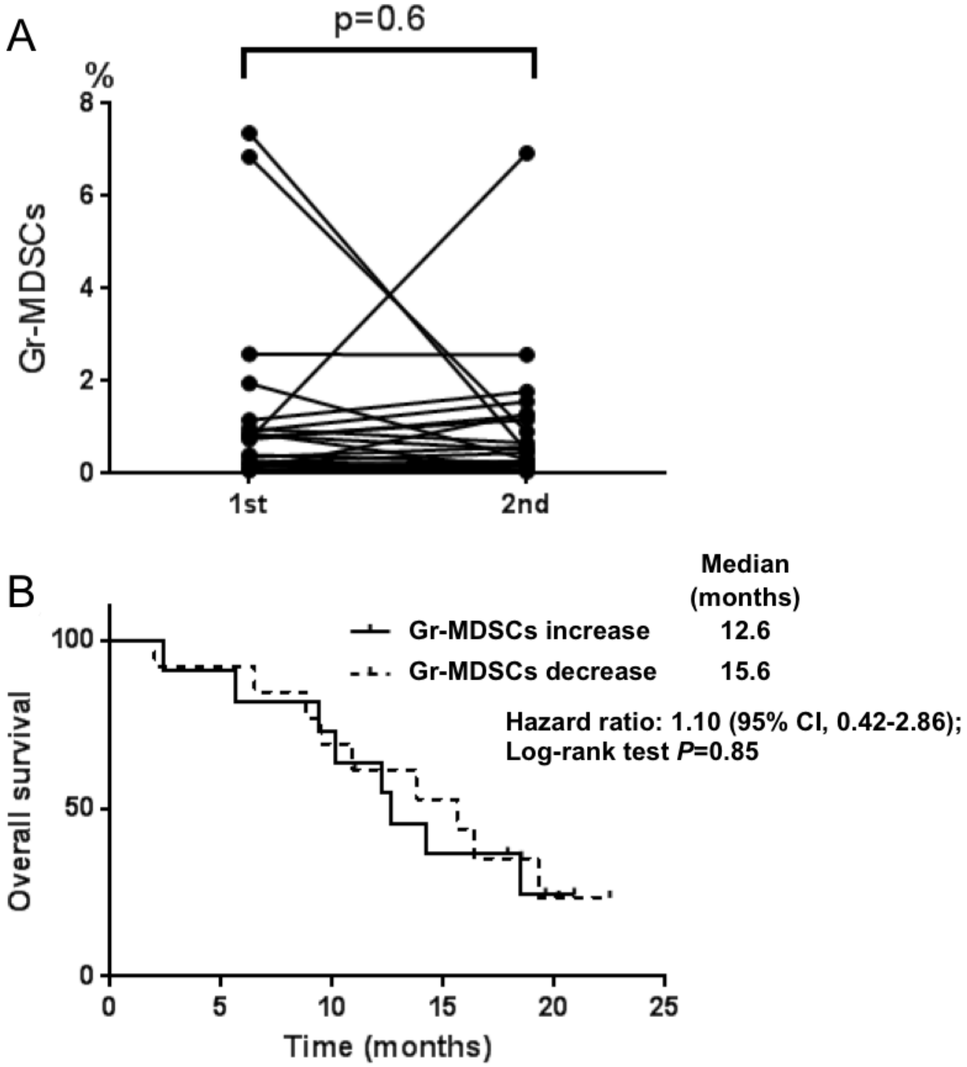

Supplementary Figure 2: (**A**) The means of %Gr-MDSCs was compared between before (1st blood collection) and after chemotherapy (2nd blood collection) using a paired-*t* test (**B**) Overall survival curves calculating using the Kaplan-Meier methods for groups classified according to the increased proportion of Gr-MDSCs from baseline versus decreased proportion of Gr-MDSCs from baseline.

Supplementary Table 1: Subsequent anti-cancer therapies

Subsequent therapy, %	Gr-MDSCs	
	Low group	High group
Patients who received ≥ 1 subsequent anti-cancer therapy	75.0	82.4
Subsequent anti-cancer therapies		
Taxan-based	50.0	71.4
Irinotecan-based	41.7	28.6
Other	8.3	0

Gr-MDSCs, granulocytic myeloid-derived suppressor cells.

Supplementary Table 2: Patient characteristics according to Gr-MDSCs subgroups

Clinical feature	No. of cases (%)	Gr-MDSCs		*P*-value
		low	high	
Total N	33	16	17	
Age				0.721
< 65	22 (66.7)	10	12	
≥ 65	11 (33.3)	6	5	
Sex				0.732
Male	15 (45.5)	8	7	
Female	18 (54.5)	8	10	
Performance status				1.000
0	10 (30.3)	5	5	
1	23 (69.7)	11	12	
Disease status				1.000
Stage IV	22 (66.7)	11	11	
Recurrence	11 (33.3)	5	6	
Histology				0.438
Intestinal type	8 (24.2)	5	3	
Diffuse type	25 (75.6)	11	14	
Target lesion				1.000
Yes	14 (42.4)	7	7	
No	19 (57.6)	9	10	
Number of metastatic sites				0.494
1	17 (51.5)	7	10	
≥ 2	16 (48.5)	9	7	
HER2 status				1.000
Positive	1 (3.0)	0	1	
Negative	32 (97.0)	16	16	
ALP				0.438
≤ 359	24 (72.7)	13	11	
> 360	9 (27.3)	3	6	
Treatment regimen				0.728
Cisplatin+S−1	20 (60.6)	9	11	
Docetaxel+Cisplatin+S−1	13 (39.4)	7	6	

Gr-MDSCs, granulocytic myeloid-derived suppressor cells; ALP, alkaline phosphatase.

Supplementary Table 3: Comparison of 8 cytokine concentrations in plasma according to Gr-MDSCs subgroups

Factors	mean ± SD (pg/mL)		*P*-value
	Gr-MDSCs		
	low	high	
IFN-γ	8.88 ± 6.20	11.18 ± 16.02	0.68
IL-1β	0.15 ± 0.22	0.09 ± 0.05	0.99
IL-4	0.009 ± 0.005	0.012 ± 0.011	0.65
IL-6	1.36 ± 1.08	3.81 ± 7.10	0.40
IL-8	12.61 ± 6.03	22.27 ± 11.72	0.01
IL-10	0.28 ± 0.14	0.48 ± 0.40	0.21
IL-12p70	0.09 ± 0.04	0.08 ± 0.05	0.48
TNF-α	2.57 ± 0.98	2.71 ± 0.76	0.42

SD, standard deviation; Gr-MDSCs, granulocytic myeloid-derived suppressor cells.

Supplementary Table 4: Association between the cytokine concentrations and progression-free survival

Factors		Hazard ratio*	95% CI	*P*-value
Cytokine values	IFN-γ	0.45	0.19–1.03	0.06
	IL-1β	1.53	0.72–3.24	0.27
	IL-4	1.71	0.77–3.78	0.19
	IL-6	2.09	1.00–4.40	0.05
	IL-8	2.66	1.23–5.75	0.01
	IL-10	1.05	0.51–2.16	0.90
	IL-12p70	0.44	0.18–1.06	0.07
	TNF-α	1.90	0.89–4.09	0.10

* Low group (<median) in each cytokine was used as a reference.

Supplementary Table 5: Association between the cytokine concentrations and overall survival

Factors		Hazard ratio*	95% CI	*P*-value
Cytokine values	IFN-γ	0.61	0.27–1.37	0.23
	IL-1β	1.83	0.80–4.18	0.15
	IL-4	1.60	0.66–3.88	0.30
	IL-6	4.55	1.87–11.1	0.0008
	IL-8	4.68	1.89–11.6	0.0009
	IL-10	1.24	0.55–2.77	0.60
	IL-12p70	0.79	0.35–1.79	0.58
	TNF-α	2.55	1.04–6.25	0.04

* Low group (< median) in each cytokine was used as a reference.
